# 17-β estradiol increases parvalbumin levels in *Pvalb* heterozygous mice and attenuates behavioral phenotypes with relevance to autism core symptoms

**DOI:** 10.1186/s13229-018-0199-3

**Published:** 2018-03-02

**Authors:** Federica Filice, Emanuel Lauber, Karl Jakob Vörckel, Markus Wöhr, Beat Schwaller

**Affiliations:** 1 Anatomy Unit, Section of Medicine, University of Fribourg, Route Albert-Gockel 1, CH-1700 Fribourg, Switzerland; 2 Behavioral Neuroscience, Faculty of Psychology, Philipps-University of Marburg, Gutenbergstraße 18, 35032 Marburg, Germany; 3 Marburg Center for Mind, Brain, and Behavior (MCMBB), Hans-Meerwein-Straße 6, 35032 Marburg, Germany

**Keywords:** ASD, Parvalbumin, 17-β estradiol, Estradiol treatment, Excitation/inhibition balance, Social behavior, Ultrasonic vocalizations

## Abstract

**Background:**

Autism spectrum disorder (ASD) is a group of neurodevelopmental disorders characterized by two core symptoms: impaired social interaction and communication, and restricted, repetitive behaviors and interests. The pathophysiology of ASD is not yet fully understood, due to a plethora of genetic and environmental risk factors that might be associated with or causal for ASD. Recent findings suggest that one putative convergent pathway for some forms of ASD might be the downregulation of the calcium-binding protein parvalbumin (PV). PV-deficient mice (PV−/−, PV+/−), as well as Shank1−/−, Shank3−/−, and VPA mice, which show behavioral deficits relevant to all human ASD core symptoms, are all characterized by lower PV expression levels.

**Methods:**

Based on the hypothesis that PV expression might be increased by 17-β estradiol (E2), PV+/− mice were treated with E2 from postnatal days 5–15 and ASD-related behavior was tested between postnatal days 25 and 31.

**Results:**

PV expression levels were significantly increased after E2 treatment and, concomitantly, sociability deficits in PV+/− mice in the direct reciprocal social interaction and the 3-chamber social approach assay, as well as repetitive behaviors, were attenuated. E2 treatment of PV+/+ mice did not increase PV levels and had detrimental effects on sociability and repetitive behavior. In PV−/− mice, E2 obviously did not affect PV levels; tested behaviors were not different from the ones in vehicle-treated PV−/− mice.

**Conclusion:**

Our results suggest that the E2-linked amelioration of ASD-like behaviors is specifically occurring in PV+/− mice, indicating that PV upregulation is required for the E2-mediated rescue of ASD-relevant behavioral impairments.

**Electronic supplementary material:**

The online version of this article (10.1186/s13229-018-0199-3) contains supplementary material, which is available to authorized users.

## Background

Autism spectrum disorder (ASD) core symptoms include impaired sociability, communication problems, and restricted or repetitive behaviors. The etiology of ASD remains still unclear, but recent advances in genetics and genomics have provided powerful tools to unraveling how mutations in certain genes might result in ASD [1]. Although a plethora of genetic and environmental risk factors are associated with ASD [2], therapeutic approaches of treating ASD subjects are rather limited and moreover, most often do not target all core symptoms [3, 4].

Recent studies suggest that the calcium-binding protein parvalbumin (PV) is downregulated in some forms of ASD. Most notably, *Shank1−/−* and *Shank3−/−* mice and offspring from mice exposed to valproic acid in utero (VPA mice), all validated mouse models for ASD, are characterized by a prominent PV reduction in ASD-associated brain regions [5, 6]. Genetically modified mice deficient for PV (PV+/− and PV−/−) display behavioral deficits relevant to all human ASD core symptoms [7]. Moreover, the number of PV-immunoreactive (PV^+^) GABAergic interneurons (hereafter termed Pvalb neurons) was reported to be decreased in several cortical areas in postmortem brains of ASD subjects [8]*.* However, since no other marker than PV had been used to identify the Pvalb neurons in that study, the decreased number of PV^+^ neurons might equally well be the result of a decreased number of Pvalb neurons or of a decrease in PV expression levels, as also acknowledged by the authors. Preliminary results on two ASD brains also did not allow to unequivocally ascribing the observed decrease of PV^+^ neurons to either mechanism [9]. Recently, RNA-seq and qRT-PCR analyses of postmortem samples of frontal and temporal cortex and cerebellum from 48 ASD individuals and 49 controls revealed that the *PVALB* and *SYT2* (synaptotagmin 2) genes were among the most strongly downregulated ones in the ASD group [10]. In the absence of clear evidence of Pvalb neuron loss in human ASD, but of confirmed decreased *PVALB* mRNA levels in human ASD individuals and additionally of PV protein levels in mouse ASD models with construct and face validity, we hypothesize that a decrease in PV levels might represent a converging pathway of ASD pathophysiology, at least in a subgroup of ASD cases.

Estrogen receptors β (ERβ) are strongly co-localized with cortical Pvalb neurons [11], and several lines of indirect evidence (reported as an increase in the number of PV^+^ neurons) indicate that PV expression might be positively modulated by 17-β estradiol (E2) [11–13]. Moreover, in the rat pituitary cell line GH3 shown to be E2 responsive, *Pvalb* was found to be one of the most E2-responsive genes [14]. E2 administration was also found to significantly increase *Pvalb* mRNA expression resulting in an augmentation in the number of PV^+^ neurons in the CA1 pyramidal cell layer of rat hippocampus [15].

In this study, we aimed to increase PV expression in PV+/− mice to possibly re-establish the state prevailing in PV+/+ mice, both with respect to PV protein levels and also behavior, for the latter applying assays with relevance to all human ASD core symptoms [16, 17]. Our approach consisted in supplementing PV+/+, PV+/−, and PV−/− pups with E2 during the early postnatal period from postnatal day (PND) 5 to 15. This time period was chosen in order to cover the critical period of sexual differentiation of neuronal circuits in rodents [18] and the previously reported developmental onset of PV expression in the rodent brain [19, 20]. Moreover, Pvalb neurons are implicated in the maturation of the cortical GABA inhibitory circuitry, including the modulation (initiation, termination) of critical developmental periods. A maturation index of Pvalb neurons proposed by Gandal et al. [21] shows a highly significant correlation between Pvalb neuron maturation status and PV expression levels. Whether this is only correlative in nature or possibly causal, i.e., PV “driving” Pvalb neuron maturation to some extent, is currently unknown. It has been shown before that E2 induces a “significant increase of parvalbumin immunoreactive neurons” in both the deep and superficial cortical layers in rat organotypic slice cultures in vitro [12]. Moreover, in vivo, estrogens have been shown to have a potential compensatory effect at behavioral levels in animal models carrying mutations in genes associated with ASD, such as the reeler heterozygous mice (rl+/−) or the *CNTNAP2−/−* zebrafish ASD model [22, 23]. However, none of these studies directly proved whether E2 treatment leads to an upregulation of PV at the transcript and/or protein levels.

In the present study, we found that E2 treatment of PV+/− mouse pups increased PV protein levels and consequently ameliorated ASD-associated social behavioral deficits and decreased repetitive behavior in later life. Thus, we provide a rationale for the positive effects of E2 on ASD-linked behavior and propose that E2 treatment should be given further attention as a potential therapeutic strategy in ASD.

## Methods

### Mouse colonies and genotyping

Mice were group-housed and maintained as described before [7]. PV-deficient mice (PV−/−; B6.Pvalb^tm1Swal^) [24] congenic with C57Bl/6J [25] were mated with C57Bl/6J wild-type mice. Heterozygous breedings were set up in order to be able to compare littermates. Day of birth was defined as PND0; genotyping and paw marking was carried out at PND2-3. Only male animals were used in this study. All experiments were performed with permission of the local animal care committee (Canton of Fribourg, Switzerland) and according to the present Swiss law and the European Communities Council Directive of 24 November 1986 (86/609/EEC).

### Estradiol treatment and determination of PV protein and *Pvalb* mRNA levels

Litters, including all genotypes, were randomly assigned to one of two experimental groups. In the first group, pups were administered with vehicle (sesame oil; 10 μl/day/pup), while the second group received 17-β estradiol (E2; Sigma-Aldrich, Buchs, Switzerland) at a dose of either 10 μg E2/day/pup (modified from [26]) or 50 μg E2/day/pup (modified from [27]) in 10 μl vehicle between PND5-15. Thus, mice of all genotypes from the same litter either received E2 or vehicle. Additional details on mouse husbandry, mouse handling, and E2 administration are reported in Additional file 1. Pups were weaned at PND22 and tested at (I) PND25 ± 1 for reciprocal social interaction and communication, (II) PND26 ± 1 for social approach in the three-chamber assay, and (III) PND31 ± 1 for repetitive, ritualistic behavior in the marble-burying test, following our previously established protocols [7, 28]. In all behavioral assays, littermate controls were included and for all experiments and analyses, experimenters were blinded with respect to genotype and treatment. For the determination of PV protein and *Pvalb* mRNA levels, PND25 was chosen, the identical time point as for the start of behavioral experiments; mice were sacrificed by cervical dislocation; and brains were collected for Western blot analyses and qRT-PCR analyses as described before [5].

### Direct reciprocal social interaction and ultrasonic vocalizations

Prior to testing, mice were socially isolated for 24 h in order to enhance the level of social motivation. To measure reciprocal social interaction behavior, pairs of juvenile mice were allowed to socially interact at PND25 ± 1 for 5 min after one mouse of the pair was habituated to the test environment for 1 min. Same-treatment/same-genotype pairs consisting of non-littermates were used. Experimental details, including measurement of ultrasonic vocalizations and behavioral analysis, have been reported before [7]. Specifically, reciprocal social interactions were recorded using a video camera. Direct reciprocal social interactions were scored and analyzed offline by an experienced observer with high reliability (inter-rater correlation coefficient: *r* = 0.902; *p* < 0.001; Pearson) using the Noldus The Observer XT 10.0 software (Noldus Information Technology, Wageningen, The Netherlands). Parameters of social behaviors included facial sniffing (sniffing the nose and snout region of the partner), anogenital sniffing (sniffing the anogenital region of the partner), following (walking straight behind the partner, keeping pace with the one ahead), push past (squeezing between the wall and the partner), crawling under/over (pushing the head underneath the partner’s body or crawling over or under the partner’s body), social grooming (grooming the partner), and being socially inactive while having social contact (lying flat or standing motionless, while maintaining close physical contact with the partner). In addition to social behaviors, non-social behaviors were measured and included rearing (number of times an animal reared on its hind legs), grooming (number of bouts of face, body, and genital grooming movements) and digging (number of bouts of digging in the bedding, pushing, and kicking it around). Parameters of social behaviors, such as anogenital sniffing, nose-to-nose sniffing, or following, were grouped together, and a mouse was scored as “engaging in a social interaction” any time those behaviors were observed. Results from reciprocal social interaction and ultrasonic vocalization assays reflect the cumulative performance of the two animals in the assay.

### Social approach behavior in the three-chamber assay

Sociability in PV+/+, PV+/−, and PV−/− mice treated or not with E2 was determined by the well-described three-chamber social approach task for each individual mouse [29]. The apparatus consisted of an open rectangular box (60 × 40 × 40 cm) divided into three chambers by retractable doors. Stranger stimulus mice were C57Bl/6J mice of the same sex and age as the test subjects. The test session began with a 10-min habituation period, with the subject mouse free to move in all the empty compartments of the chamber; in the meanwhile, a new unfamiliar stranger mouse was habituated (10 min) to a wire cup; an identical empty wire cup was used as novel object. After the 10-min habituation period, the test subject was briefly confined to the center chamber and the novel object was placed in one of the side chambers, while the novel mouse was placed on the other side. The location of the novel object and the novel mouse were alternated between the left and right chambers across test subjects to avoid a side preference bias. After both stimuli were positioned, the two side doors were simultaneously lifted and the subject mouse had access to all three compartments for 10 min. The time spent in each compartment and entries into each one and the time spent exploring the novel mouse or the empty cup were manually scored by an observer blinded to the mouse genotype/treatment using two stopwatches. Exploration of an enclosed mouse or of the empty wire cup was scored positive, when the test mouse was oriented with the head towards the cup within a 2-cm distance between the head of the mouse and the cup, or when climbing on the cup. Between tests, the apparatus was cleaned with 0.1% acetic acid and water.

### Marble-burying test

The marble-burying test consisted of introducing items that a mouse can bury during a set period of time; mice with an ASD-associated behavior often tend to engage in a higher degree of digging (burying) than what is observed in controls [28]. Mice were individually placed in Plexiglas type III cages containing 5-cm deep clean bedding with 20 ceramic marbles (14 mm diameter) arranged in 5 × 4 evenly spaced rows as described before [30]. Test duration was 30 min. Marbles were considered buried, if more than half of a marble was covered with bedding. A greater number of buried marbles was considered as an indication for increased repetitive behavior.

### Statistical analysis

For analysis of direct reciprocal social interaction and ultrasonic vocalizations in juvenile mice, two-way ANOVAs with the between-subject factors genotypes (PV+/+, PV+/−, PV−/−) and treatment (vehicle vs. E2) plus the covariate age of subject mice (PND) were calculated. In order to test whether differences in direct reciprocal social interaction behavior and the emission of ultrasonic vocalizations emerged over time during testing, ANOVAs for repeated measurements with the same between-subject factors plus the covariate age of subject mice and the within-subject factor test duration were performed. Paired *t* tests were used to compare the likelihood of the occurrence of a social behavior in response to a social behavior, and one-sample *t* tests for comparisons with chance levels. Three-way ANOVAs for repeated measurements with the within-subject factor preference (mouse vs. object) and the between-subject factors genotype and treatment (vehicle vs. E2) were used to analyze social approach behavior in the three-chamber assay. Marble-burying behavior was analyzed using a two-way ANOVA with the between-subject factors genotype and treatment (vehicle vs. E2). ANOVAs were followed by LSD post hoc analysis or paired/unpaired Student’s *t* tests when appropriate. Western blot analysis was performed, and protein levels were compared between genotypes and treatments using planned Student’s *t* tests. For all the experiments, a *p* value < 0.05 was considered statistically significant. Data were analyzed using IBM SPSS Statistics 22 (Armonk, USA) and GraphPad Prism software (San Diego, USA).

## Results

Here, we demonstrate that E2 upregulates PV expression in PV+/− mice leading to an amelioration of the ASD-related phenotypes previously described for PV-deficient (PV+/−, PV−/−) mice [7]. Conversely, in most assays, E2 treatment of PV+/+ mice unexpectedly provoked ASD-like behaviors, whereas in PV−/− mice, E2 had no significant effect in all behavioral tests carried out.

### PV upregulation via 17-β estradiol administration

**Fig. 1:**
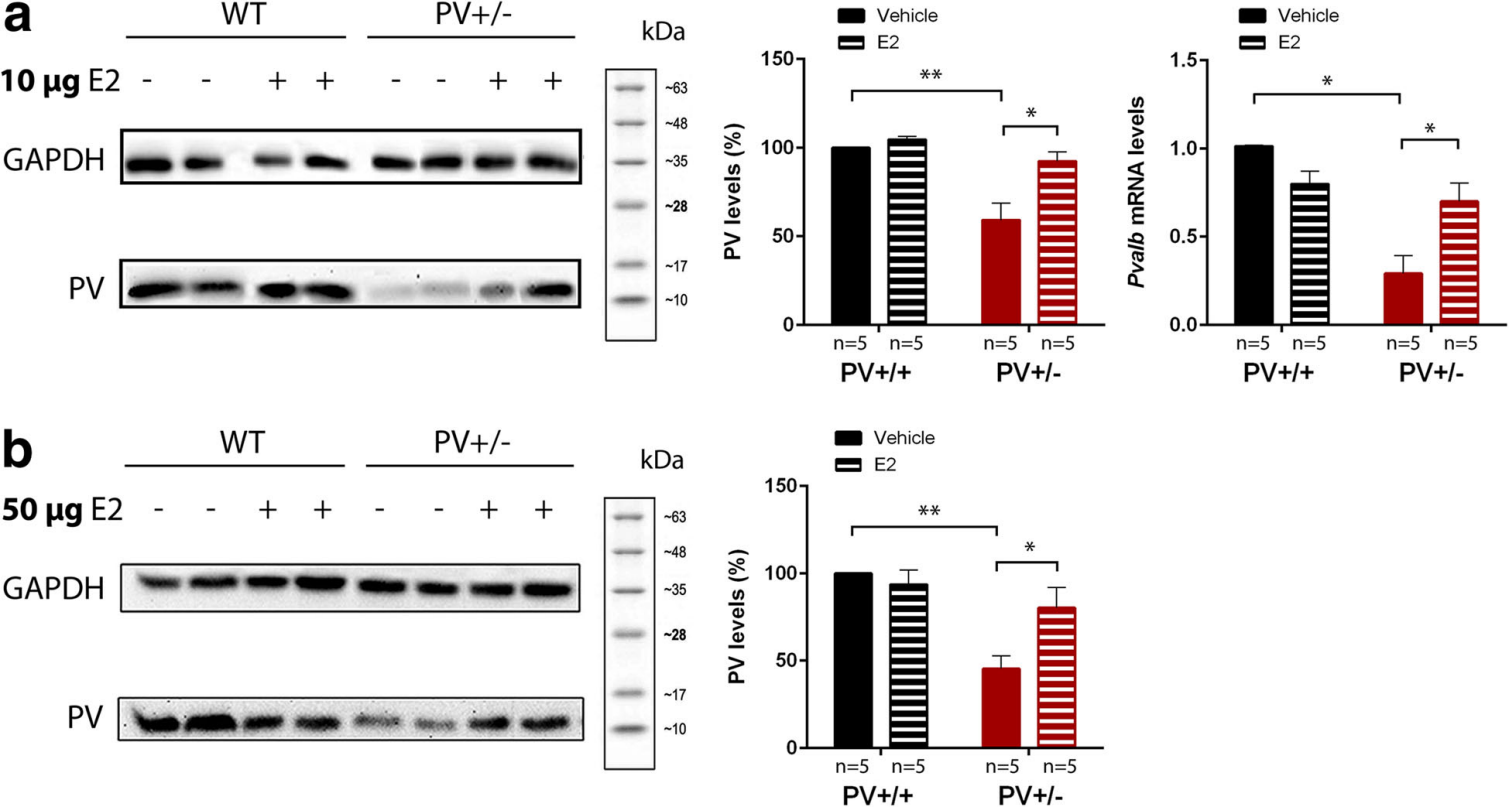
Western blot analysis and RT-qPCR of forebrain samples from PV+/+ and PV+/− mice either vehicle-treated (−) or E2-treated (+) from PND5-15. **a** Left: representative Western blots of PV (M_r_: 12 kDa) and GAPDH (M_r_: 35 kDa; normalization signal) are shown. Middle: quantitative analysis of PV signals in mice treated with 10 μg E2/day/pup. Data are obtained from three independent experiments and are shown as mean ± SEM. Results are expressed as a percentage of normalized PV levels as measured in vehicle-treated PV+/+ samples (set as 100%). GAPDH was used for the normalization of the PV signals. Right: RT-qPCR analysis of forebrain samples of PV+/+ and PV+/− mice. *Pvalb* mRNA levels were normalized to 18S mRNA levels and expressed as fold change. Data from three independent experiments were pooled and are shown as mean ± SEM. **b** Representative Western blots (left) and quantitative analysis of PV signals from samples derived from mice treated with 50 μg E2/day/pup (right). PV signal quantification was done as in **a**. Asterisks represent *p < 0.05, **p < 0.01, respectively

Based on direct and indirect evidence linking PV expression levels with estradiol [12, 22], we investigated whether E2 upregulates PV expression in PV+/− mice. PV levels in PV+/− forebrain samples were significantly lower, i.e., in the order of 50% compared to PV+/+ samples (*p* = 0.003; Fig. 1), in line with previous findings [5, 31, 32] and PV was completely absent in PV−/− mouse extracts (not shown). Both E2 treatments (10 or 50 μg/day) did not significantly affect PV expression levels in PV+/+ animals at PND25. Importantly, E2 treatment of PV+/− mice in the period from PND5 to PND15 resulted in a persistent increase in PV determined at PND25 (10 μg E2: *p* = 0.011; 50 μg E2: *p* = 0.041; Fig. 1). Since no significant differences in the degree of recovery of PV expression were observed in PV+/− animals treated with 10 or 50 μg E2, the lower dose was chosen for all behavioral experiments.

To evaluate how well *Pvalb* mRNA and protein expression levels correlate after E2 administration, we performed RT-qPCR analysis. *Pvalb* mRNA levels of E2-treated (10 μg) PV+/− mice were significantly increased, to a similar extent as PV protein levels (Fig. 1a), in comparison with PV+/− vehicle-treated mice (*p* = 0.041). This is suggestive of a modulation of PV via E2 at the transcriptional level, in line with previous studies [14]. No changes were seen in PV+/+ animals after E2 administration (Fig. 1a).

### Direct reciprocal social interaction and ultrasonic vocalizations

**Fig. 2:**
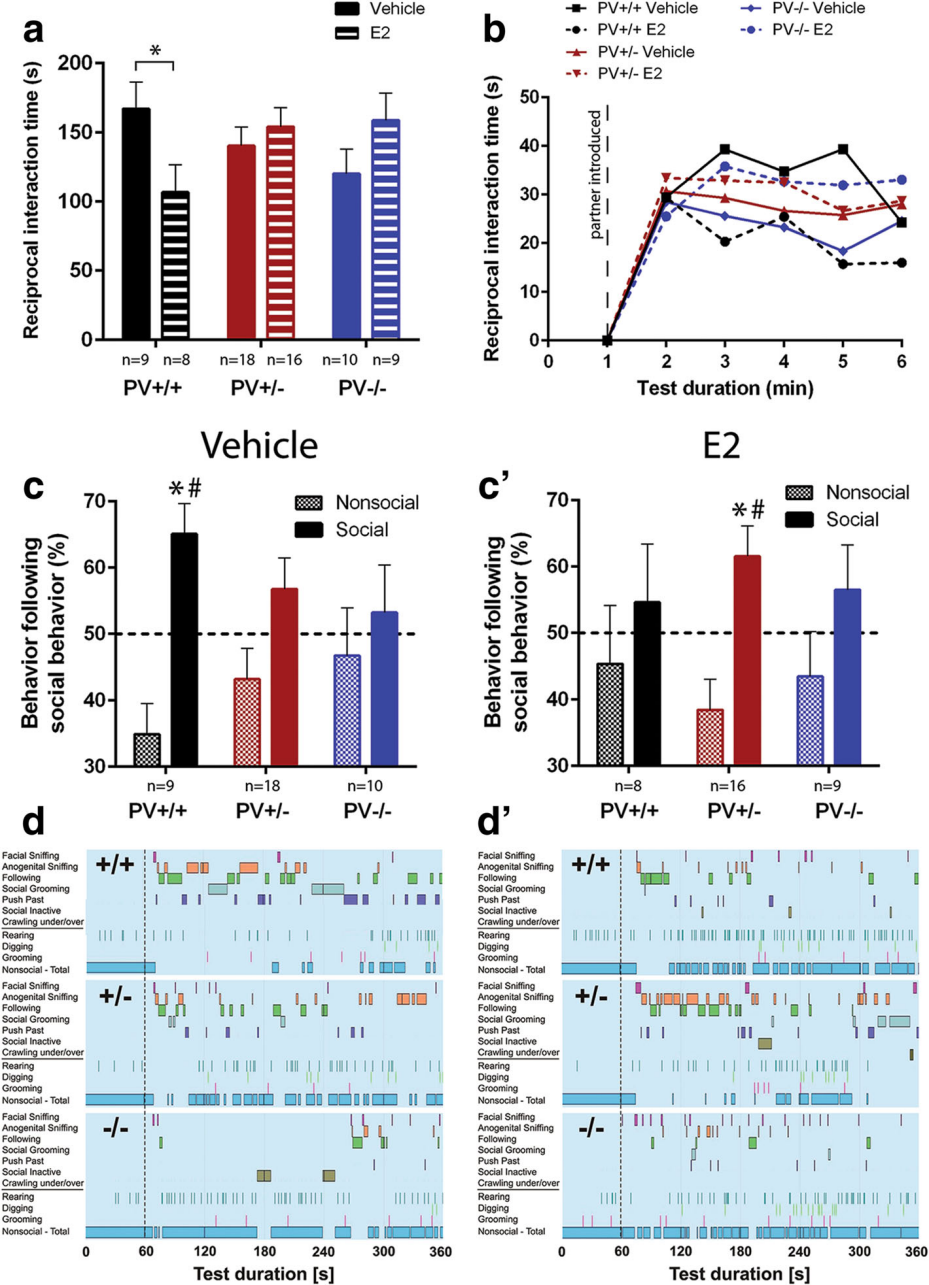
Reciprocal social interaction test. **a** The total social interaction time displayed by pairs of the same genotype during the 5-min social interaction period in the cohorts of PV+/+, PV+/−, and PV−/− mice, vehicle-treated or E2-treated. Asterisk represents *p < 0.05. **b** Time course of time spent in social interaction per 1-min bin (dashed line indicates introduction of partner mouse). Data are presented as mean ± SEM. **c** Percentage of non-social vs. social behavior following social behavior in PV+/+ wild-type littermate control mice, PV+/− heterozygous mice, and PV−/− mice treated with vehicle (**c**) or E2 (**c’**). The dashed line indicates 50% chance level. Black bar: social; striped bar: non-social. Asterisk represents *p* < 0.05 vs. non-social. Hashtag represents *p* < 0.05 vs. 50% chance level. **d** Representative ethograms of social and non-social behavior displayed during juvenile reciprocal social interactions by a PV+/+ wild-type littermate control mouse, a PV+/− heterozygous mouse, and a PV−/− null mutant mouse treated with vehicle (**d**; left) or E2 (**d’**; right)

Direct reciprocal social interaction was tested in PND25 ± 1 juvenile mice. The time spent in reciprocal social interactions was visibly affected by E2 treatment in a genotype-dependent manner (genotype: *p* = 0.805; treatment: *p* = 0.848; genotype x treatment: *p* = 0.044; Fig. 2a). In vehicle-treated PV−/− mice, we observed a tendency to engage less in reciprocal social interactions (− 28% interaction time) compared to PV+/+ littermate controls (*p* = 0.077), while the decrease was found insignificant in PV+/− mice (*p* = 0.269). In a previous study, a similar decrease in social interaction time had been observed; however, the overall effect was slightly larger (− 36%) and also significant in the PV+/− mouse group [7]. While in the group of E2-treated PV+/+ mice, the time spent in reciprocal social interactions was significantly decreased compared to the vehicle-treated PV+/+ mice (*p* = 0.037); no differences in interactions were observed in E2-treated PV+/− and PV−/− mice in comparison to corresponding vehicle-treated mice (*p* = 0.475 and *p* = 0.147, respectively). However, there was a notable tendency of increase in comparison to the E2-treated PV+/+ littermate controls (*p* = 0.056 and *p* = 0.073, respectively), an effect that was mostly attributable to a “worsening” of the E2-treated PV+/+ mice. Along the 5-min examination period, the time spent in social interactions was rather constant in all groups, with a trend for a decrease towards the end (Fig. 2b). When analyzing the social behavior repertoire in detail, its richness and reciprocal character were found to be strongly dependent on genotype (Fig. 2c). While vehicle-treated PV+/+ mice displayed a significant preference for engaging in another social behavior following a previous one in ~ 65% of the cases (~ 35% for non-social behavior; *p* = 0.011 vs. chance level), no such preference was seen in vehicle-treated PV+/− and PV−/− mice, with social behaviors following in ~ 57 and ~ 53% of cases, respectively (*p* = 0.165 and *p* = 0.665 vs. chance level, respectively). The genotype-dependent effect size was approximately of the same magnitude as observed in our previous study (compared to Fig. 1d in [7]). In E2-treated mice, however, a different pattern emerged (Fig. 2c’). Most remarkably, in E2-treated PV+/− mice, a social behavior was followed by another one in ~ 62% of cases (*p* = 0.024 vs. chance level), almost reaching the situation prevailing in “normal,” i.e., vehicle-treated PV+/+ mice. While E2 treatment had no prominent effect in PV−/− mice, with social behaviors following in ~ 57% of cases (*p* = 0.359 vs. chance level), E2 treatment had detrimental effects in PV+/+ mice, with social behaviors following in only ~ 55% of cases (*p* = 0.614 vs. chance level). Representative ethograms of mouse pairs are depicted in Fig. 2d, d’. There was no evidence for genotype × treatment interaction effects on non-social behaviors, including rearing, grooming, and digging behavior (Fig. 2d, d’, all *p* values > 0.100). The different social behavior components (e.g., facial sniffing, following, etc.) as shown in the ethograms (Fig. 2d, d’) were statistically analyzed in more detail, i.e., for each behavior component independently and results are presented in Additional file 1. Only in one of the social behaviors (social grooming), we observed a weak genotype × treatment interaction (*p* = 0.03) indicating that behaviors grouped as “social behavior” represent general effects that cannot be fragmented into meaningful individual social behavior components.

**Fig. 3:**
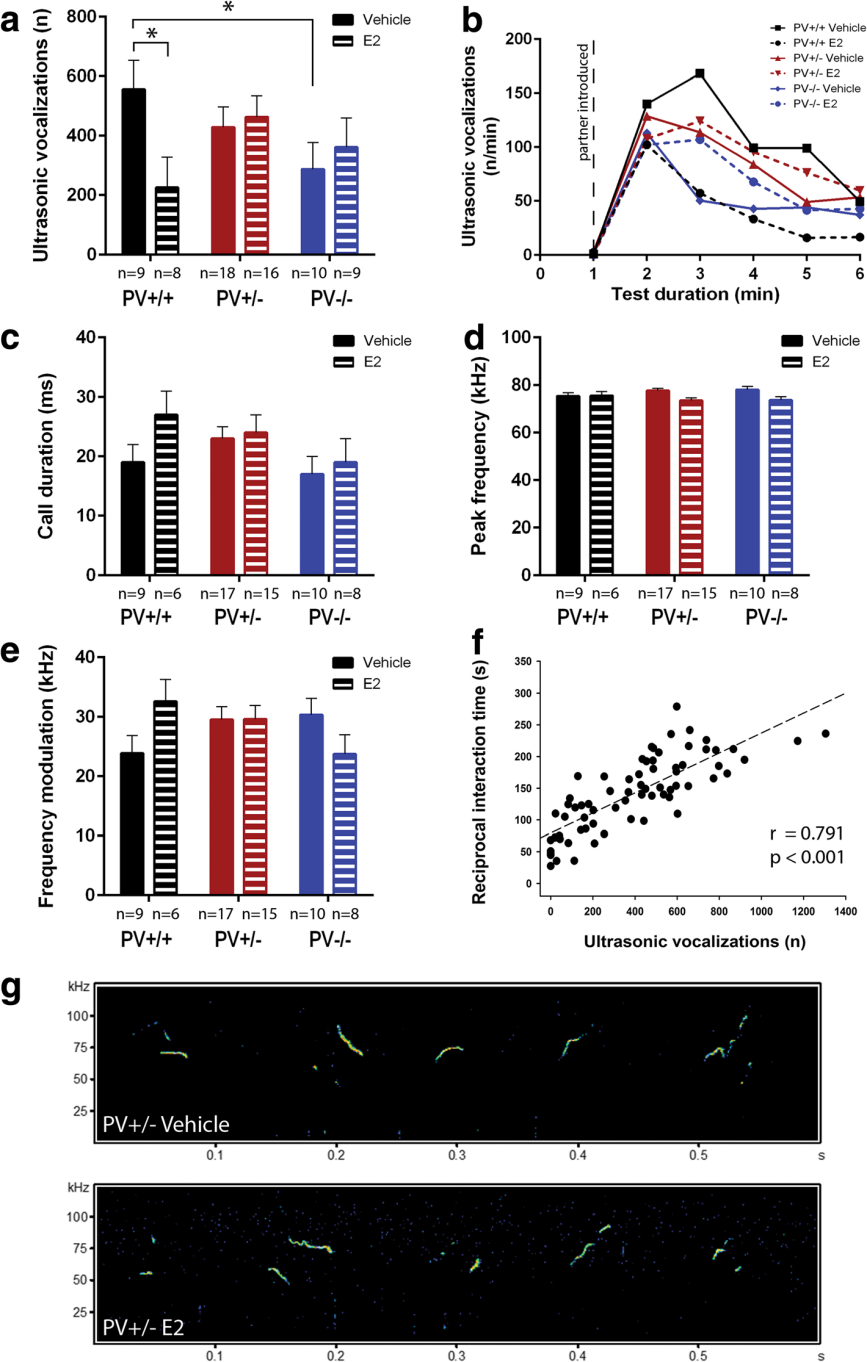
Analysis of ultrasonic vocalizations between PV+/+, PV+/−, and PV−/− mouse pairs. Irrespective of treatment, all genotypes displayed similar call emission patterns. **a** Total number of calls emitted during the 5-min social interaction period; asterisks represent *p < 0.05. **b** Time course for the number of ultrasonic vocalizations emitted for each 1-min time bin across the 5-min social interaction period, plus 1 min habitation (dashed line indicates introduction of partner mouse). Data are presented as mean ± SEM. **c** Average call duration, **d** peak frequency and **e** frequency modulation of emitted calls during the 5-min social interaction period. **f** Correlation between ultrasonic vocalizations and time spent in social interaction for each animal. **g** Representative spectrograms of ultrasonic vocalizations emitted during juvenile reciprocal social interactions of a vehicle-treated (upper traces) and an E2-treated (lower traces) PV+/− mouse are shown

Ultrasonic vocalization emission rates during reciprocal social interactions tended to be weakly affected by E2 treatment in a genotype-dependent manner (genotype: *p* = 0.355; treatment: *p* = 0.298; genotype × treatment: *p* = 0.090; Fig. 3a). Numbers of vocalizations were significantly decreased in vehicle-treated PV−/− mice compared to the corresponding PV+/+ group (*p* = 0.047; Fig. 3a). An intermediate phenotype was observed in vehicle-treated PV+/− mice, although the decrease in vocalization numbers compared to PV+/+ was not significant (*p* = 0.303), likely due to the large variations between individual mice of all genotype and treatment groups (Fig. 3a). Of note, genotype differences in vehicle-treated mice were less prominent than observed previously [7], possibly as the result of extensive/prolonged mouse handling during daily E2 or vehicle treatments (see discussion). Call numbers were significantly decreased in E2-treated PV+/+ compared to vehicle-treated PV+/+ mice (*p* = 0.025), but call numbers were essentially unchanged in E2- or vehicle-treated PV+/− and also PV−/− groups (*p* = 0.723 and *p* = 0.582, respectively) (Fig. 3a). The time course of vocalizations, i.e., the highest numbers of calls occurring within the first 2 min after the addition of the second mouse, followed by a gradual decline (Fig. 3b) was similar as reported before [7].

There was no evidence for genotype × treatment interaction effects on acoustic call features, including call duration (Fig. 3c), peak frequency (Fig. 3d; all *p* values > 0.100) and frequency modulation (*p* = 0.083; Fig. 3e). Irrespective of genotype and treatment, the emission of ultrasonic vocalizations was highly positively correlated with the time spent in reciprocal social interaction (*r* = 0.791, *p* < 0.001; Fig. 3f). Representative spectrograms are depicted in Fig. 3g for a pair of vehicle-treated or E2-treated PV+/− mice; the other four groups of mice revealed very similar patterns of calls (not shown).

### Social approach behavior in the three-chamber assay

**Fig. 4:**
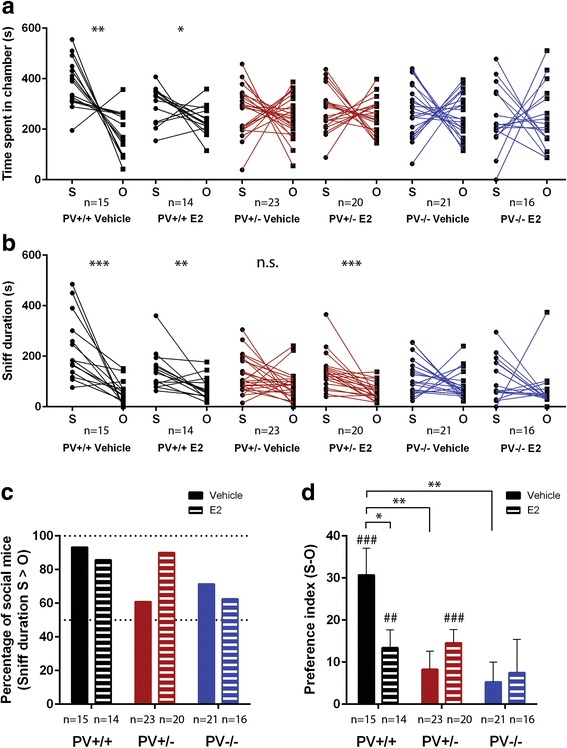
E2 administration differently affected sociability of PV+/+, PV+/−, and PV−/− mice tested in the three-chamber assay. **a** Paired graphs of time spent in the chamber with the novel mouse (S) or the object (O) during the 10-min social interaction period plotted for each mouse; a negative slope (S > O) is characteristic of a “social” mouse. **b** Sniffing/exploration time spent close to the subject mouse, i.e., within 2 cm from the stranger/empty wired cup. **c** Percentage of “social” mice defined as animals with sniff duration time (S) > (O) during the 10-min test phase. **d** Preference index (stranger vs. object sniffing time divided by the total exploration time in the compartments containing S and O) fold change. Values close to “0” indicate no preference for the stranger mouse. Asterisks represent *p < 0.05; **p < 0.01; ***p < 0.001; n.s.: not significant. Hashtags represent significant preference for the stranger mouse over the object ^##^*p* < 0.01; ^###^*p* < 0.001

In the often applied three-chamber test [29], sociability is defined as preference for a novel mouse (subject; S) over a novel object (empty wire cup; O). Preference is typically assessed by means of time spent in chambers containing the novel mouse versus the novel object. This preference was clearly genotype-dependent (preference: *p* = 0.002; preference × genotype: *p* = 0.014; Fig. 4a; data are additionally presented as bar graphs in Additional file 1: Figure S3A). Vehicle-treated PV+/+ mice showed a strong preference for the chamber containing the novel mouse compared to the one with the novel object (*p* = 0.001), while no such preference was observed in vehicle-treated PV+/− and PV−/− mice (all *p* values > 0.100). Mice from the latter two groups (PV+/−, PV−/−) showed an almost equal interest in the two chambers. E2 treatment slightly decreased the S/O ratio in PV+/+ mice; however, mice still spent significantly more time in the S chamber (*p* = 0.046). E2 treatment of PV+/− and PV−/− mice did not affect the time spent in the S and O compartments (preference × treatment: *p* = 0.165; preference × genotype × treatment: *p* = 0.335).

More relevant than a mouse’s simple presence in the S- and O-containing chamber of the cage and thus representing a more sensitive measure of sociability is the measurement of sniff duration, i.e., the time spent within a 2-cm distance from the wire cup [16, 33]. With this measurement, genotype differences were even more prominent (preference: *p* < 0.001; preference × genotype: *p* = 0.009; Fig. 4b; data presented as bar graphs in Additional file 1: Figure S3B). In congruence with the results shown in Fig. 4a, vehicle-treated PV+/+ mice spent significantly more time in sniffing the novel mouse than the novel object (*p* < 0.001), while vehicle-treated PV+/− mice showed a much weaker (if any; *p* = 0.057) and PV−/− mice no such preference for the novel mouse (*p* = 0.126). The more sensitive sociability-related measure “sniff duration” further revealed that the preference for the novel mouse over the novel object was clearly modulated by E2 treatment in a genotype-dependent manner (preference × treatment: *p* = 0.265; preference × genotype × treatment: *p* = 0.042; Fig. 4b and Additional file 1: Figure S3B). E2 treatment of PV+/− mice considerably increased the duration of sniffing the novel mouse, resulting in a substantial preference towards it (*p* < 0.001), to levels observed in E2-treated or vehicle-treated PV+/+ mice. In the E2-treated PV+/− group, the percentage of mice with S > O sniff time rose to 90%, similar to values in the vehicle- (14 out of 15; 93.3%) and E2-treated (12 out of 14; 85.7%) PV+/+ group (Fig. 4c), indicative of an almost complete E2-mediated rescue in otherwise less social PV+/− mice. Such a change was not observed in E2-treated PV−/− mice (*p* = 0.342). E2-treated PV+/+ mice still displayed a preference for the novel mouse (S) over the novel object (O) (*p* = 0.008); however, such a preference was notably decreased. Of note, the results obtained in the three-chamber social approach assay were robust insofar, as the result pattern did not change depending on whether climbing on the cup was included in the sniffing analysis or not. In fact, the time that mice spent on top of either the stimulus (S) or object (O) cup closely resembled the picture observed for sniff time, simply on a much smaller time scale (compare Additional file 1: Figures S3B and S4A).

In an additional exploratory approach, this genotype effect was also evident when comparing the often used preference index, defined as the numerical difference between time spent exploring the targets (subject vs. object, S-O) divided by the total time spent in the two compartments containing either target, as described previously [34, 35]. A clear preference for the novel mouse was observed in vehicle-treated PV+/+ mice (*p* < 0.001), while such a preference was very weak for vehicle-treated PV+/− mice (*p* = 0.070) and not observed in PV−/− mice (*p* = 0.277), resulting in significant group differences (*p* = 0.005 and *p* = 0.002 vs. PV+/+, respectively; Fig. 4d). E2 treatment significantly increased the preference index in PV+/− mice (*p* < 0.001), while this was not the case for E2-treated PV−/− mice (*p* = 0.356). There was still a significant, however, smaller (compared to vehicle-treated PV+/+) preference for the novel mouse in E2-treated PV+/+ mice (*p* = 0.008). In line with the results on reciprocal social interactions (Fig. 2a), sociability tested in the social approach assay was again decreased in E2-treated PV+/+ mice compared to vehicle-treated PV+/+ mice (Fig. 4d; *p* = 0.034), supporting the adverse effect of E2 treatment in “healthy” PV+/+ mice.

To exclude the possibility that the observed differences in preference might be the result of impaired or reduced locomotor activity in the six groups, the number of entries into the two chambers was determined and found to be unchanged (preference: *p* = 0.843; preference × genotype: *p* = 0.201; preference × treatment *p* = 0.818; preference × genotype × treatment: *p* = 0.693). However, general locomotor activity was affected (genotype: *p* = 0.003; treatment: *p* = 0.896; genotype × treatment: *p* = 0.009). In fact, under vehicle conditions genotypes did not differ (*p* = 0.765), yet under E2 treatment conditions, genotypes differed (*p* = 0.001) and PV+/− mice displayed more entries than PV+/+ and PV−/− mice (*p* = 0.001 and *p* = 0.006, respectively), possibly reflecting their more vigorous attempts to establish social contact (Additional file 1: Figure S3C).

### Marble-burying test

**Fig. 5:**
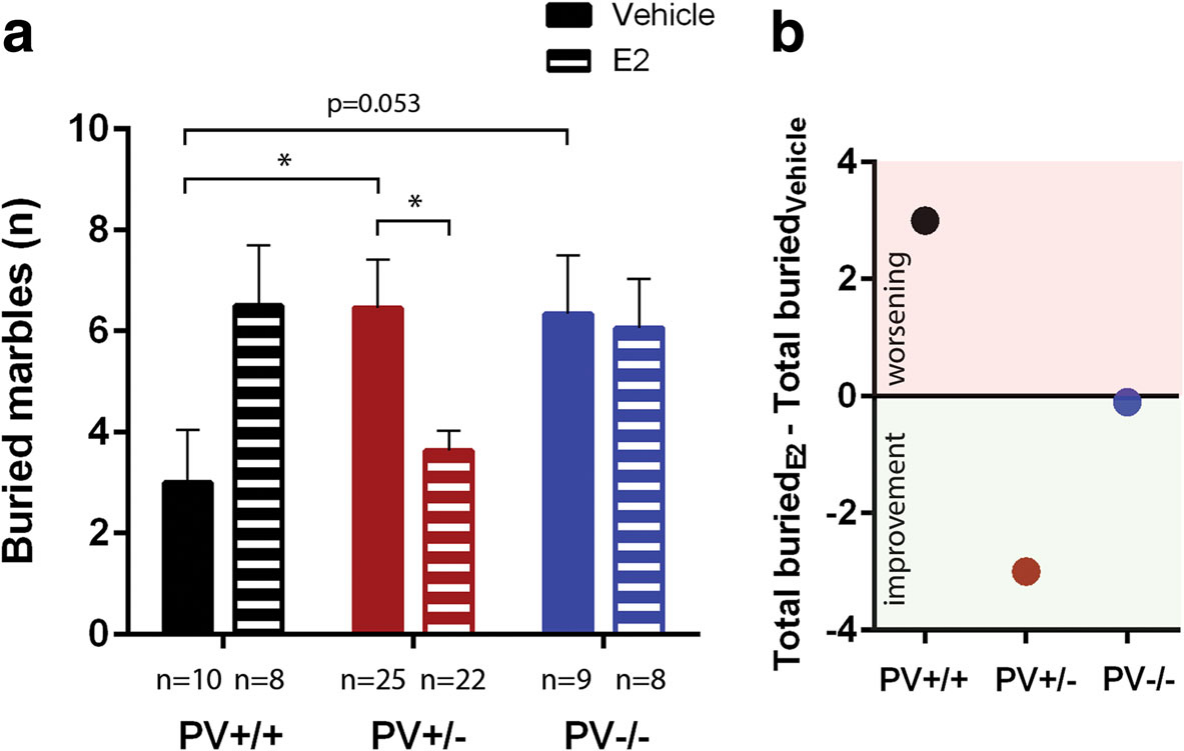
Performance of PV+/+, PV+/−, and PV−/− mice in the marble-burying test. **a** Total number of buried marbles in vehicle-treated and E2-treated mice; asterisks represent *p < 0.05. **b** Numerical difference within the same genotype groups between E2-treated and vehicle-treated mice. Negative values below “0” indicate attenuation of repetitive, ritualistic behavior; values above “0” indicate an E2-mediated increase of marble-burying behavior, i.e., an increase in ASD-associated behavior

To test repetitive behavior, we performed the marble-burying test [28]. Marble burying was visibly affected by E2 treatment in a genotype-dependent manner (genotype: *p* = 0.471; treatment: *p* = 0.982; genotype x treatment: *p* = 0.009; Fig. 5a). Vehicle-treated PV+/− and PV−/− mice buried more marbles compared to the corresponding PV+/+ mice (*p* = 0.046 and *p* = 0.053, respectively), in support of increased repetitive behavior in mice with reduced or absent PV levels [7]. While E2 treatment of PV+/+ mice increased their marble burying behavior (*p* = 0.051), E2-treated PV+/− mice buried less marbles than their corresponding vehicle-treated PV+/− group (*p* = 0.011). No E2 effects were observed in PV−/− mice (*p* = 0.832). Also the marble-burying test hints towards a common effect of E2 administration: an increase (worsening) in ASD-associated behaviors in PV+/+ and an attenuation (improvement) of ASD-associated behaviors in PV+/− mice (Fig. 5b), closely approaching the behavior of vehicle-treated PV+/+ mice.

## Discussion

PV-deficient mice (PV+/−, PV−/−) represent a new genetic mouse model of ASD, displaying a behavioral phenotype characterized by impairments in social interaction, communication, and perseveration [7]. In these mice, PV expression levels are downregulated/absent, while the number of Pvalb neurons is unaltered compared to PV+/+ mice [5]. The same holds true for *Shank1−/−*, *Shank3−/−*, and VPA mice [6, 36], three well-established mouse models for ASD [28, 37–41]. Thus, we addressed the question whether the direct upregulation of PV via neonatal administration of E2 might ameliorate or even abrogate the ASD phenotype displayed by male juvenile PV+/− mice. E2 administration in PV+/− mice increased PV expression to levels closely approaching vehicle-treated PV+/+ mice; at the behavioral level, this coincided with an attenuation of the ASD-related phenotypes. Most evidently, the richness and reciprocal character of E2-treated PV+/− mice was substantially increased, as quantified by a preference for engaging in another social behavior following a previous one. Such a preference was repeatedly reported in mice displaying intact sociability, including, but not limited to [42], i.e., control (PV+/+) mice [7]. Consistently, a preference for engaging in another social behavior following a previous one was also seen in vehicle-treated PV+/+ mice in the present study, but not in PV+/− and PV−/− mice. Importantly, such a preference was also evident in E2-treated PV+/− mice, almost reaching the situation of vehicle-treated PV+/+ mice, while E2 treatment had no positive effect in PV−/− mice. This suggests that our recently established approach to quantify the preference for engaging in another social behavior following a previous one offers a new and unique possibility to assess treatment effects on the richness and the heterogeneity of the direct reciprocal social behavior repertoire displayed by juvenile mice. However, while this appears a promising strategy, our approach to quantify the preference for engaging in another social behavior is new and its validity and potential in revealing treatment effects thus would merit an independent dedicated validation study. There, comparing several ASD mouse models with known social deficits would reveal whether this parameter might be helpful for the identification/validation of an ASD-like phenotype in mice.

Supporting the results from the direct reciprocal social interaction test, the “sniff duration” time, being the most sensitive parameter in the three-chamber social approach assay, revealed a pro-social effect of E2 treatment exclusively in PV+/− mice. The lack of social preference seen in vehicle-treated PV+/− and PV−/− mice is consistent with their altered reciprocal social interaction behavior when compared to PV+/+ mice. Only in PV+/− mice, E2 treatment considerably increased the sniffing S/O ratio of individual mice; 90% showed a preference for the novel mouse vs. 61% in vehicle-treated animals, indicative of an E2-mediated increase in sociability in otherwise “non-social” PV+/− mice.

Communication deficits in PV+/− and PV−/− mice observed before [7], were also seen in vehicle-treated PV+/− and PV−/− mice, but were only significant in PV−/− mice. E2 treatment had no effect in PV+/− and PV−/− mice but caused a reduction of call number in PV+/+ mice. The absence of a treatment effect on ultrasonic vocalizations might be attributable to the prolonged postnatal handling during E2/vehicle administration. In agreement, the average number of calls in vehicle-treated PV+/+ mice (556) was much higher than in the previous study where mice were subjected to substantially less handling (average 355 in PV+/+ mice; [7]). It is known that neonatal handling increases call emission in pups, which in turn, serves to increase maternal care [43, 44]. The stressful context associated with extensive handling [45] is the most likely explanation for the increased production of ultrasonic vocalizations in all genotypes compared to our previous study [7], possibly masking genotype-dependent effects of E2. As assessment of repetitive behaviors, we performed the marble-burying test; E2-treated PV+/− animals performed better (i.e., buried less marbles) than vehicle-treated PV+/− mice, consistent with previous findings showing that female sex hormone levels modulate marble-burying behavior [46, 47]. Moreover, the overall rather low numbers of marbles buried is likely the result of the juvenile age (PND31), when mice were tested; a significant age-dependent increase in marble-burying behavior from PND24 (≈ 10%) to PND40 (≈ 30% marbles buried) has been observed before [46].

Taken together, our results confirm that E2 can ameliorate the investigated ASD-like symptoms, in line with previous findings supporting a role for E2 as a promising candidate in amelioration of ASD-related phenotypes [22, 23]. In particular, we observed a ≈ “rescue effect” in sociability tests and amelioration of repetitive behaviors selectively in E2-treated PV+/− mice, but not in the PV+/+ and PV−/− groups. It is important to remark that E2 had no significant effect on PV protein levels in juvenile PV+/+ and PV−/− mice; thus, any effect observed in these mice after E2 treatment excludes PV being the effector of the putative changes. Nevertheless, E2 might have effects per se, previously evidenced by its role in modulation of social behavior [48] and modulation of neural circuits via direct or indirect activation of multiple downstream signaling pathways [49]. In contrast to primates, where brain circuits are primarily masculinized by the action of androgens, it is presumed that in the brain of rodents, such masculinizing effects are mainly mediated by estrogens [50]. Repetitive exposure to estrogens in neonatal PV+/+ males from PND5 to PND15 might thus induce an “extreme male brain,” according to the theory proposed by Baron-Cohen [51]. This might explain the “anti-social” effect of E2 treatment in PV+/+ mice (reduced ultrasonic vocalizations, decreased sociability) as well as the increase of repetitive behaviors (higher number of buried marbles compared to controls).

Our results indicate that E2 is sufficient to partially reverse ASD-relevant behaviors, if it is associated with a reinstatement of PV levels similar to the ones seen in PV+/+ mice. The E2-mediated re-establishment of PV levels is assumed to also restore the electrophysiological phenotypes associated with the absence of PV, i.e., stronger short-term facilitation, increased excitability, increased regularity of fast-spiking interneuron firing in PV−/− mice [52]. The presumed functional restoration of the Pvalb-neuron containing networks is then likely to also restore the excitation/inhibition (E/I) ratio in E2-treated PV+/− mice, the E/I balance assumed to be a key factor in ASD [53, 54]. Our results in male PV+/+ mice, however, indicate that E2 treatment in a situation defined by a balanced E/I ratio actually leads to a “worsening” in communication and sociability tasks and an increase in repetitive behaviors.

An optimal range of PV circuit function in the insular cortex has been proposed before, where either an imbalance towards more excitation or more inhibition was shown to impair multisensory integration in several ASD mouse models including *Shank3* and *Mecp2* knockout mice [55]. In the latter, the trajectory of functional maturation of Pvalb neurons in the primary visual cortex is accelerated upon vision onset (advanced onset and closure of the critical period), based on higher expression levels of PV and GAD67, vGAT, perineuronal nets, and enhanced GABA transmission among Pvalb neurons at PND15 [56]. In the ASD knockout mouse model for BMP/RA-inducible neural-specific protein 1 (Brinp1−/−), the higher density of PV^+^ neurons in the somatosensory cortex and medial hippocampus in adult mice, without indication of altered neuronal proliferation and apoptosis during embryonic development, is indicative of increased PV levels associated with the ASD phenotype [57]. Thus, putative E2-mediated alterations in the trajectory of PV expression and/or associated Pvalb network maturation in E2-exposed PV+/+ mice might be responsible for the appearance of an ASD phenotype possibly linked to accelerated maturation.

The identification of targets for the E2-mediated negative effects on the behavior of PV+/+ mice requires further investigations. It remains to be investigated whether E2-mediated increases in PV in other ASD mouse models, including *Shank1*, *Shank2*, and *Shank3* knockouts [58, 59] or VPA mice consequently ameliorates ASD-associated behaviors. If successful, E2 treatment and/or other means of selective PV upregulation might represent a new avenue towards improvement, i.e., “normalization” of behavior in human ASD.

## Conclusions

Our study confirms that PV-deficient mice (PV+/− and PV−/−) show a discernable ASD-like phenotype. ASD-associated behaviors (decreased social interaction, augmented repetitive behaviors) displayed by PV+/− mice are strongly ameliorated, if PV expression is restored close to PV+/+ levels via early postnatal E2 administration. In PV−/− mice, where E2 treatment has obviously no effect on PV levels, the ASD-like phenotype persists. Unexpectedly, in E2-treated PV+/+ mice, ASD-associated behaviors arise. Our results point towards a key role of PV upregulation in PV+/− mice with regard to the amelioration of ASD-like behaviors. An increase in PV expression mediated by E2 in other ASD mouse models with reduced PV levels might also improve ASD-like behavior and possibly represent a point of convergence facilitating the search for new therapeutic approaches in ASD.

## Additional file

Additional file 1: **Supplemental information on experimental details**. **Figure S1 A**) Details on mouse husbandry and **B**) selection of the tested mice. **Figure S2** Methodological details on mouse handling, E2 administration and mouse weights. **Figure S3** Results from 3-chamber social approach assay presented as bar graphs (**A** and **B**) and number of entries in S and O chamber (**C**). **Figure S4** Analysis of cup climbing time during the 3-chamber social approach assay. **Table S1** Component analysis of behaviors scored in social reciprocal interaction. (DOCX 3208 kb)

## Abbreviations

ASD: Autism spectrum disorders
E2: 17-β estradiol
ERβ: Estrogen receptor β
FSI: Fast-spiking interneurons
GABA: Gamma aminobutyric acid
GAD67: Glutamic acid decarboxylase 67
O: Object
PND: Postnatal day
PV: Parvalbumin
rl: Reeler
S: Subject
SYT2: Synaptotagmin 2
vGAT: Vesicular GABA transporter
VPA: Valproic acid
