# Supplementary material for: 17-β estradiol increases parvalbumin levels in Pvalb heterozygous mice and attenuates behavioral phenotypes with relevance to autism core symptoms
Source: Mol Autism. 2018 Mar 2;9:15. doi: 10.1186/s13229-018-0199-3 (PMC5833085; doi:10.1186/s13229-018-0199-3)
Supplement: Supplementary file 1 — Supplemental information on experimental details. Figure S1 A) Details on mouse husbandry and B) selection of the tested mice. Figure S2 Methodological details on mouse handling, E2 administration and mouse weights. Figure S3 Results from 3-chamber social approach assay presented as bar graphs (A and B) and number of entries in S and O chamber (C). Figure S4 Analysis of cup climbing time during the 3-chamber social approach assay. Table S1 Component analysis of behaviors scored in social reciprocal interaction. (DOCX 3208 kb) [file 13229_2018_199_MOESM1_ESM.docx]

**Supplemental Information**

**1) Mouse husbandry and selection of the tested mice**

Due to the challenging criteria for matching the mice in the reciprocal social interaction test (only male pups, same age, same genotype, same treatment, non-littermates), a rather large number of pups were excluded from the study. In total, pups derived from 57 different litters were used in the study, resulting in an average of 2.45 pups per litter that were included in the tests (ranging from 1-6; suppl. Fig. 1A). The low number of pups used from the same litter also excluded the observed effects on behavior being the result of a “litter effect”. The use of many animals from the same litter was previously reported to cause a severe bias resulting in litter effects [1]. Obviously in litters, where only one or two mice fulfilled all criteria, there was a prevalence of PV+/- mice (theoretically present within a litter with 2-fold higher probability than either PV+/+ or PV-/- mice (suppl. Fig. 1B).

**A B**

**
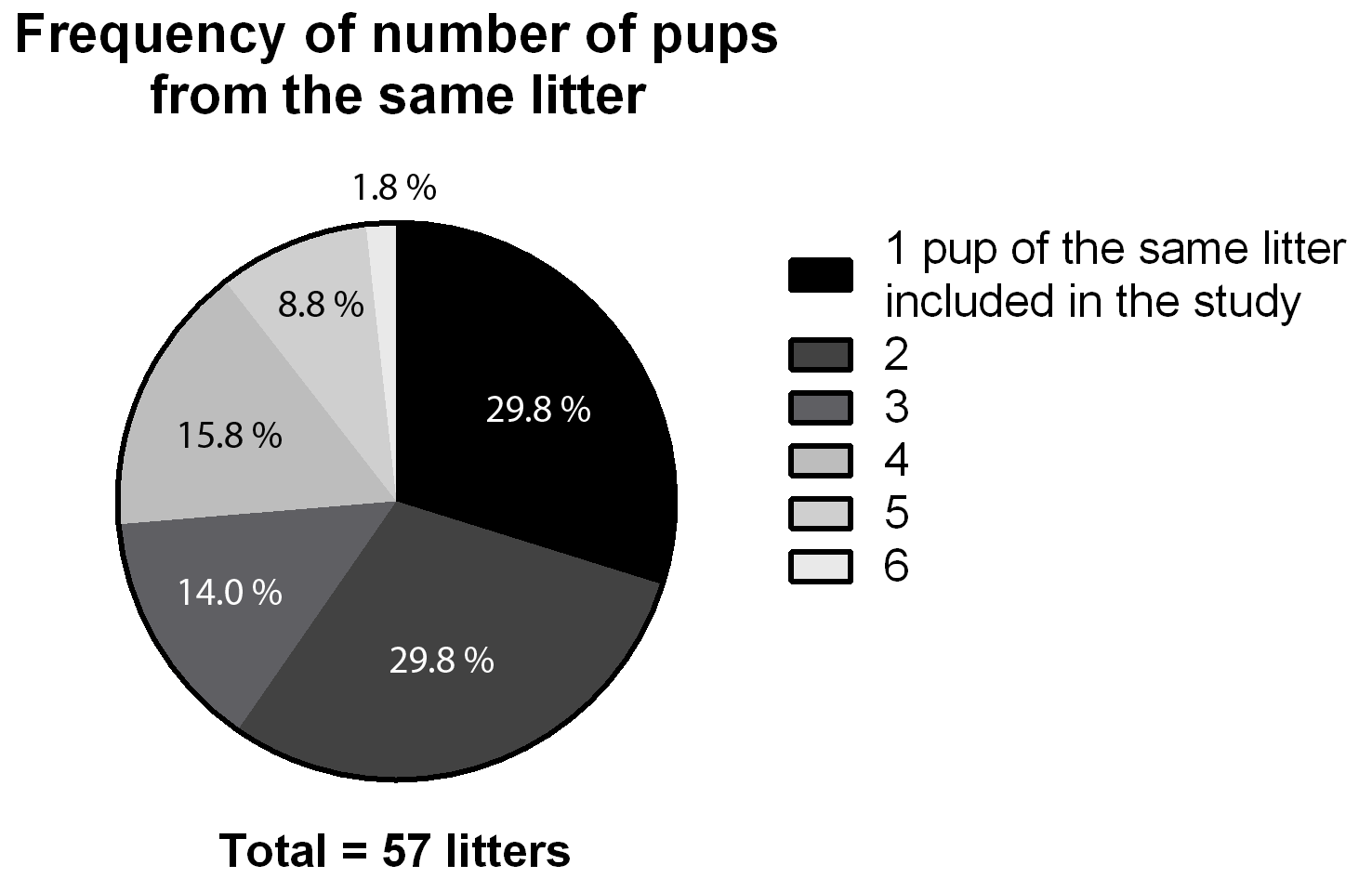
**


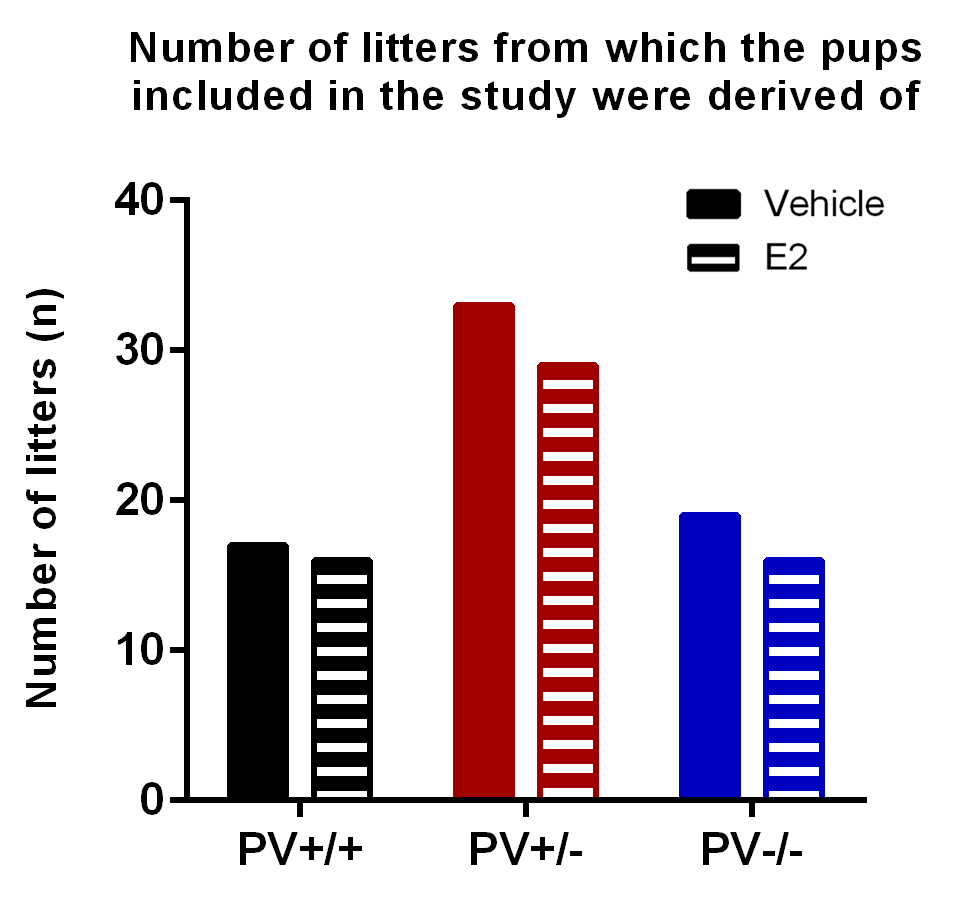


**Suppl. Fig. 1 A)** Approximately 60% of mice used in experiments derived from a litter, where only one pup (black) or two pups (dark grey) from the same litter were used. **B)** PV+/- mice are derived from a higher number of litters than either PV+/+ or PV-/- mice. This further decreases a litter bias of the E2-treatment effect in the PV+/-group.

**2) Mouse handling and E2 administration**

Details of the treatment were adapted from the study of Patisaul [27], where no weight adjustment was applied for the treatment of the pups at this age, i.e. all pups received the same dose (10 µg/day or 50 µg/day E2), irrespective of weight. Daily weighing was assumed to represent additional stress for the pups at this age. In our study, pups from the same litter were randomly assigned to both groups (E2-treated and vehicle-treated), thus excluding a litter bias. All mice received the same volume (10 µl) of sesame oil (vehicle) containing E2 only in one of the experimental groups. Before treatment, all pups were removed from the cage and put on a clean tissue paper. Pups were gently grabbed at random; female pups were returned to the cage and in male pups 10 µl of sesame oil with or without E2 were administered orally using a pipette. Placing the pipette in a pup’s mouth stimulates the suckling response of the pup resulting in the swallowing of the whole dose, generally within 5-10 s. Pups were held ventral side up to increase their suckling behavior [2].

Mice were weighted after each behavioral test. Statistical analysis of the weight after the first behavioral test (P25±1) showed no difference with respect to weight between genotypes, treatments or both (suppl. Fig. 2).

**A B**


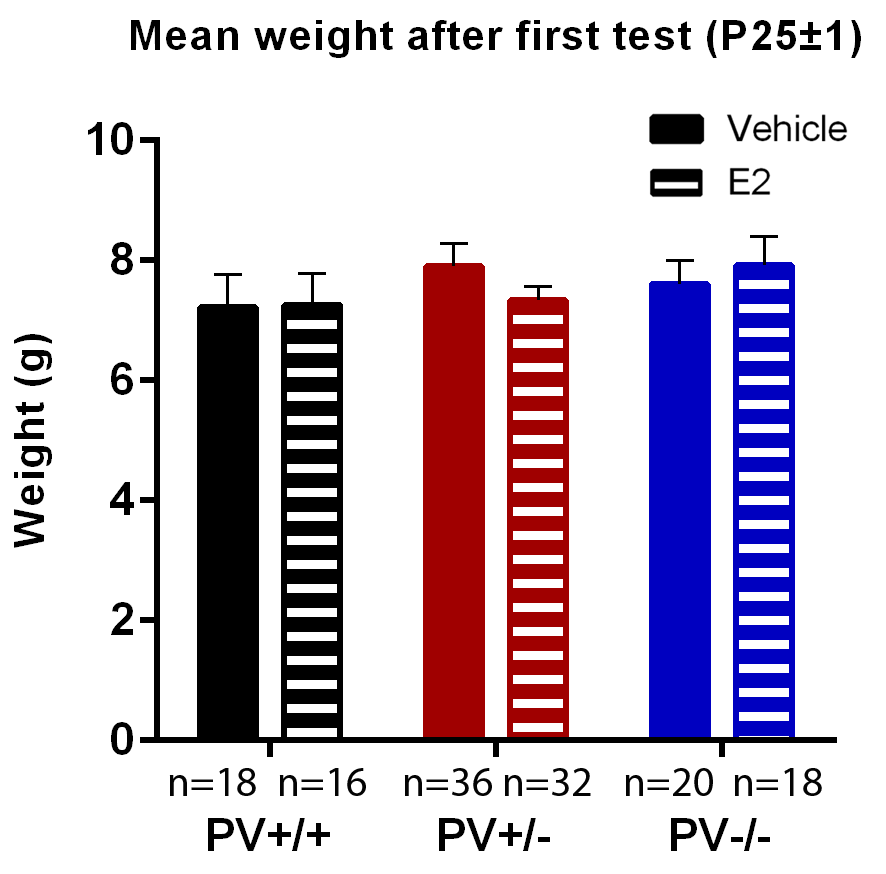

**Suppl. Fig. 2 A)** Average weight of mice at PND25±1 did not differ between genotypes (PV+/+, PV+/-, PV-/-) and treatment groups (E2, vehicle); all comparisons (genotype, treatment and genotype x treatment) did not reveal statistically significant differences; all p-values >0.100 **B)** The weight distribution histogram showed a Gaussian shape (red curve) with a mean of 7.58 g at PND25±1.

**3) Component analysis of behaviors scored in social reciprocal interaction**

The individual components of behavior as shown in Fig. 2D and 2D’ were analyzed separately with the aim to “fine-grain” the behavioral analysis. Although individual components showed significant differences with respect to genotype (“following”: p=0.03), treatment (“crawling over/under”: p=0.02) and genotype x treatment (“social grooming”: p =0.03), no consistent picture emerged (suppl. Table 1). A comparison of the results with the ones from the previous study (Ref. [7] in main text) revealed no significant genotype-dependent differences, with a slight trend with respect to the component “following” (p=0.07). Altogether this indicates that the individual components of behaviors grouped as “social behaviors” are not reliable enough to be interpreted as individual measures. Thus, we observed basically a "random" distribution of significant differences, as one would expect from any large number of dependent variables. Only by combining several lower reliability components into the parameter “Social behaviors” we were able to uncover the true genotype and/or treatment effects (Fig. 2A, Fig. 2D & 2D’). Of note the parameter “Social behavior following social behavior” proved to be the most robust parameter to reveal genotype and treatment effects.

**4) Results from 3-chamber social approach** **assay presented as bar graphs**

**Suppl. Fig. 3 A)** Bar graphs of time spent in the chamber with the novel mouse (S) or the object (O) during the 10-min social interaction period (mean ± SEM). Asterisks represent *p<0.05; **p<0.01; ***p<0.001. **B)** Sniffing/exploration time spent close to the subject mouse, i.e. within 2 cm from the stranger/empty wire cup. **C)** Number of entries into the two side compartments (containing S and O) of the chamber during the 10-min test phase. Under E2 treatment conditions, PV+/- mice showed significantly more entries in the side chambers compared to either PV+/+ or PV-/- mice. ** p<0.01 for both comparisons.

**
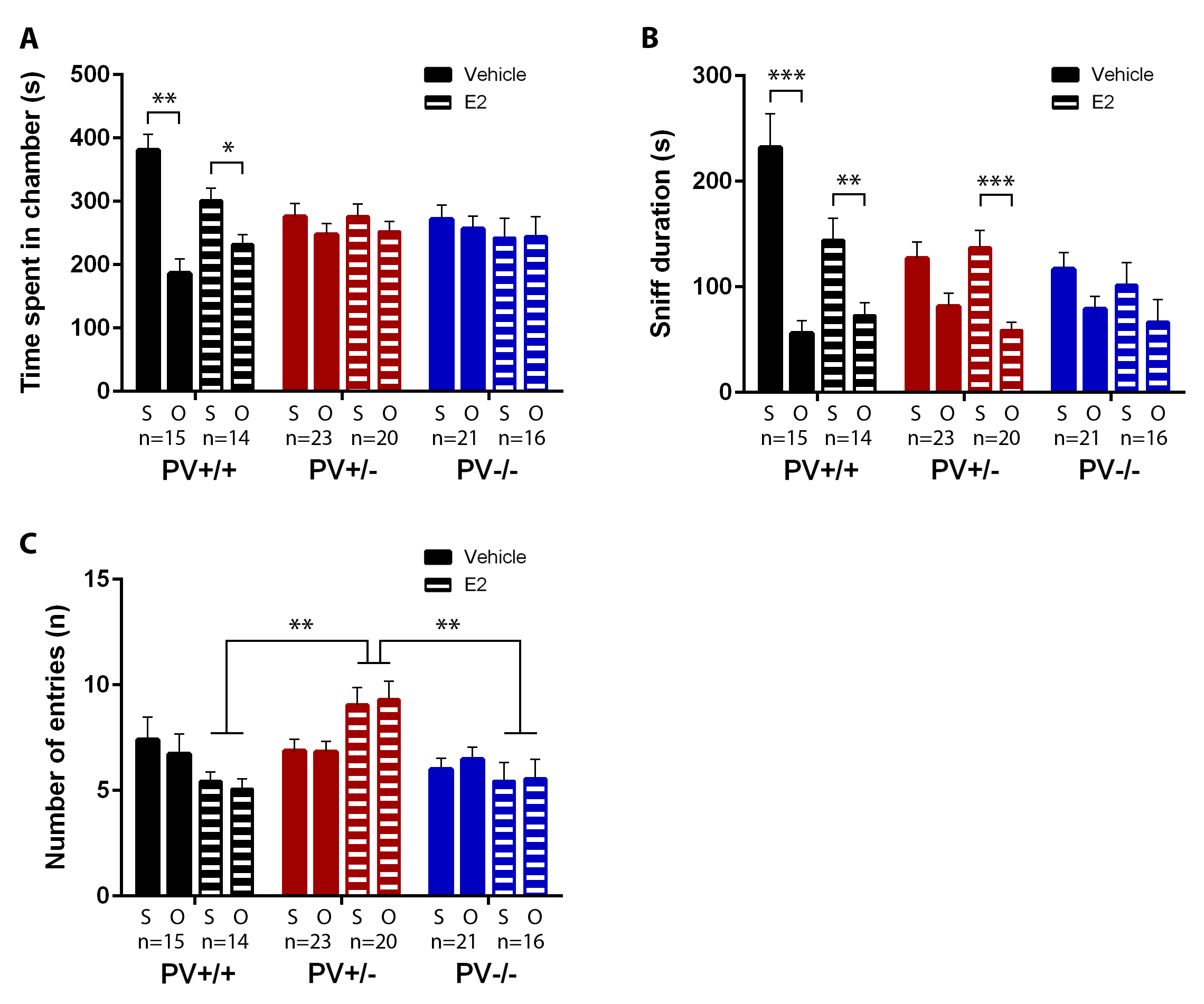
**

**5) Analysis of cup climbing during the 3-chamber social approach assay**

**Suppl. Fig. 4 A)** Bar graphs of time spent on top of the cup with the novel mouse (S) or the object (O) during the 10-min social interaction period (mean ± SEM). **B)** Bar graph of the percentage of the time spent on the cup (total sniffing time = 100 %). **C)** Percentage of mice that climbed on either cup for each group.

**
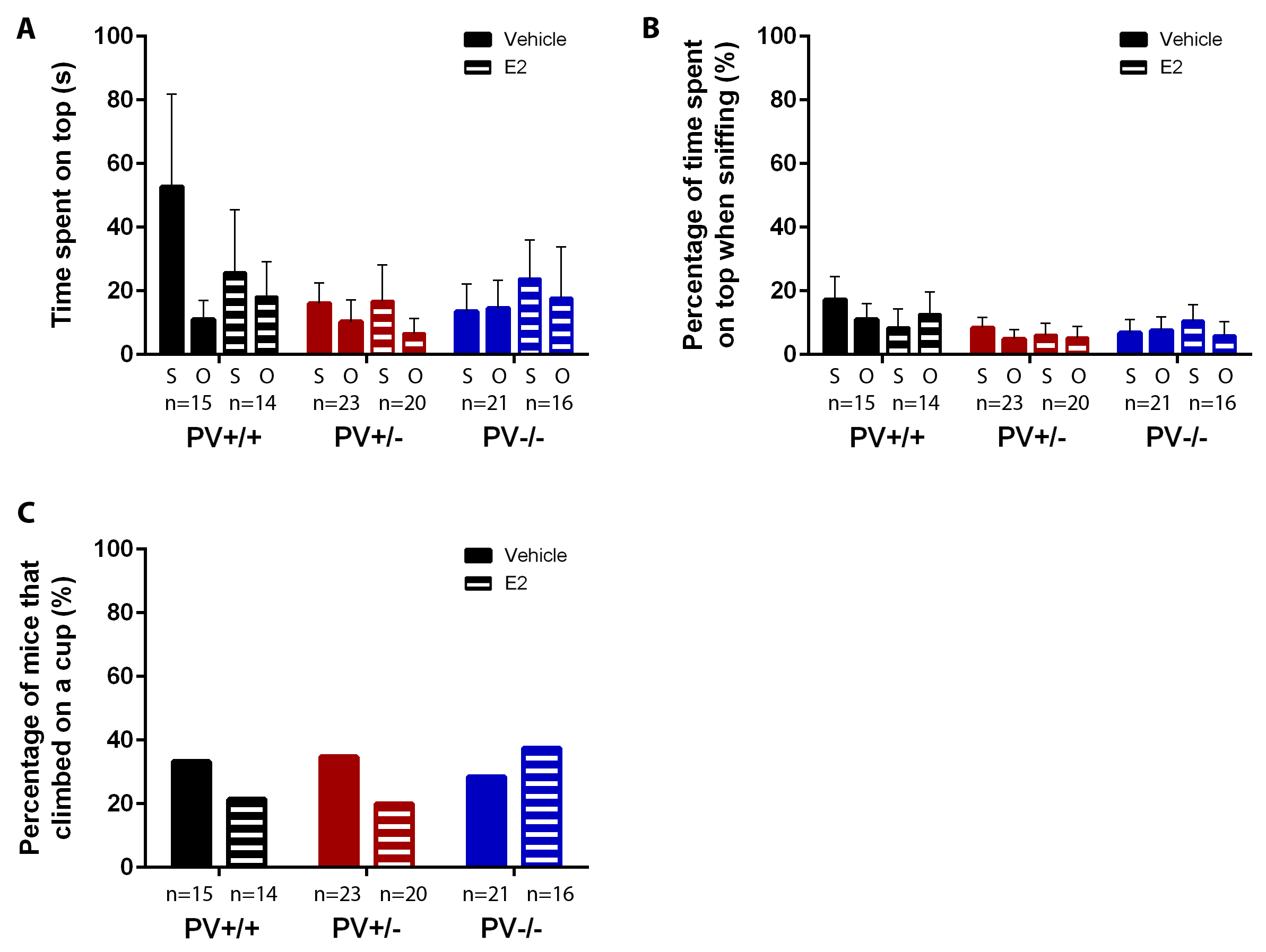
**

**Supplemental References**

1. Lazic SE, Essioux L: **Improving basic and translational science by accounting for litter-to-litter variation in animal models**. *BMC Neurosci* 2013, **14**:37.

2. Cimafranca MA, Davila J, Ekman GC, Andrews RN, Neese SL, Peretz J, Woodling KA, Helferich WG, Sarkar J, Flaws JA *et al*: **Acute and chronic effects of oral genistein administration in neonatal mice**. *Biol Reprod* 2010, **83**(1):114-121.

**Suppl. Table 1**

**Individual behavior components recorded during the reciprocal interaction test (representative data shown in Fig. 2D & 2D’)**

Significant differences are boxed in light green, trends (0.05 < p < 0.10) are boxed in light yellow

| **Results from current study** | |  |  |  |  |  |  |  |  |
| --- | --- | --- | --- | --- | --- | --- | --- | --- | --- |
|  |  |  |  |  |  |  |  |  |  |
| Behavior | PV+/+ Ctrl. | PV+/+ E2 | PV+/- Ctrl. | PV+/- E2 | PV-/- Ctrl. | PV-/- E2 | Sign.genotype | Sign. treatment | Sign. genotype x treatment |
| Facial sniffing | 14.09 ± 4.92 | 15.53 ± 5.13 | 14.28 ± 3.43 | 15.87 ± 3.56 | 14.11 ± 4.53 | 23.84 ± 4.95 | p= .60 | p= .24 | p= .59 |
| Anogenital sniffing | 73.44 ± 12.71 | 48.13 ± 13.26 | 49.13 ± 8.87 | 58.06 ± 9.21 | 47.62 ± 11.71 | 68.89 ± 12.79 | p= .80 | p= .86 | p= .20 |
| Following | 38.37 ± 7.98 | 19.27 ± 8.33 | 37.05 ± 5.57 | 47.54 ± 5.79 | 22.02 ± 7.36 | 28.77 ± 8.03 | p= .03 | p= .91 | p= .13 |
| Social grooming | 18.21 ± 3.79 | 2.91 ± 3.96 | 6.72 ± 2.65 | 7.87 ± 2.75 | 9.75 ± 3.50 | 14.04 ± 3.82 | p= .34 | p= .24 | p= .03 |
| Push past | 11.48 ± 3.55 | 8.43 ± 3.70 | 16.03 ± 2.48 | 13.57 ± 2.57 | 10.3 ± 3.27 | 6.84 ± 3.57 | p= .10 | p= .25 | p= .99 |
| Crawling over/under | -0.24 ± 1.26 | 1.19 ± 1.20 | 3.04 ± 0.84 | 0.95 ± 0.87 | 3.15 ± 1.11 | 0.64 ± 1.21 | p= .32 | p= .02 | p= .91 |
| Socially inactive | 10.44 ± 4.64 | 12.8 ± 4.84 | 14.21 ± 3.24 | 10.33 ± 3.36 | 13.34 ± 4.27 | 15.95 ± 4.66 | p= .77 | p= .91 | p= .62 |
|  |  |  |  |  |  |  |  |  |  |
| Data presented are estimated marginal means ± SEM (age as covariate) | | | |  |  |  |  |  |  |
|  |  |  |  |  |  |  |  |  |  |
| **Results from Wöhr et al. (2015), Transl. Psych.** | | |  |  |  |  |  |  |  |
|  |  |  |  |  |  |  |  |  |  |
| Behavior | PV+/+ | PV+/- | PV-/- | Sign. genotype |  |  |  |  |  |
| Facial sniffing | 7.59 ± 1.52 | 7.5 ± 1.44 | 7.91 ± 1.33 | p= .98 |  |  |  |  |  |
| Anogenital sniffing | 19.34 ± 4.47 | 13.82 ± 4.22 | 16.78 ± 3.92 | p= .67 |  |  |  |  |  |
| Following | 54.89 ± 8.08 | 35.61 ± 7.63 | 28.59 ± 7.08 | p= .07 |  |  |  |  |  |
| Social grooming | 12.5 ± 4.14 | 9.61 ± 3.91 | 8.99 ± 3.63 | p= .81 |  |  |  |  |  |
| Push past | 12.72 ± 2.34 | 9.49 ± 2.21 | 9.67 ± 2.05 | p= .54 |  |  |  |  |  |
| Crawling over/under | 1.53 ± 1.01 | 2.8 ± 0.96 | -0.04 ± 0.89 | p= .13 |  |  |  |  |  |
| Socially inactive | 40.25 ± 6.57 | 21.61 ± 6.2 | 22.89 ± 5.76 | p= .09 |  |  |  |  |  |
|  |  |  |  |  |  |  |  |  |  |
| Data presented are estimated marginal means ± SEM (age as covariate) | | | |  |  |  |  |  |  |
